# Clonal evolution in therapy-related neoplasms

**DOI:** 10.18632/oncotarget.14509

**Published:** 2017-01-05

**Authors:** Emiliano Fabiani, Giulia Falconi, Luana Fianchi, Marianna Criscuolo, Tiziana Ottone, Laura Cicconi, Stefan Hohaus, Simona Sica, Massimiliano Postorino, Antonino Neri, Marta Lionetti, Giuseppe Leone, Francesco Lo-Coco, Maria Teresa Voso

**Affiliations:** ^1^ Department of Biomedicine and Prevention, Universita’ Tor Vergata, Rome, Italy; ^2^ Department of Hematology, Universita’ Cattolica S. Cuore, Rome, Italy; ^3^ Department of Clinical Sciences and Community Health, Università degli studi di Milano, Italy

**Keywords:** therapy-related neoplasms, clonal evolution, NGS, mutation

## Abstract

Therapy-related myeloid neoplasms (t-MN) may occur as a late effect of cytotoxic therapy for a primary malignancy or autoimmune diseases in susceptible individuals. We studied the development of somatic mutations in t-MN, using a collection of follow-up samples from 14 patients with a primary hematologic malignancy, who developed a secondary leukemia (13 t-MN and 1 t-acute lymphoblastic leukemia), at a median latency of 73 months (range 18-108) from primary cancer diagnosis.

Using Sanger and next generation sequencing (NGS) approaches we identified 8 mutations (*IDH1* R132H, *ASXL1* Y591*, *ASXL1* S689*, *ASXL1* R693*, *SRSF2* P95H, *SF3B1* K700E, *SETBP1* G870R and *TP53* Y220C) in seven of thirteen t-MN patients (54%), whereas the t-ALL patient had a t(4,11) translocation, resulting in the *KMT2A/AFF1* fusion gene. These mutations were then tracked backwards in marrow samples preceding secondary leukemia occurrence, using pyrosequencing and a NGS protocol that allows the detection of low variant allele frequencies (≥0.1%).

Somatic mutations were detectable in the BM harvested at the primary diagnosis, prior to any cytotoxic treatment in three patients, while they were not detectable and apparently acquired by the t-MN clone in five patients.

These data show that clonal evolution in t-MN is heterogeneous, with some somatic mutations preceding cytotoxic treatment and possibly favoring leukemic development.

## INTRODUCTION

Therapy-related myeloid neoplasms include myelodysplastic syndromes (MDS) and acute myeloid leukemia (AML) occurring as a late effect of chemo- and/or radiotherapy for a primary malignancy or an autoimmune disease and have been included in the WHO classification of acute leukemia [1–3]. Although the majority of t-MN are of myeloid lineage, therapy-related acute lymphoblastic leukemia (t-ALL) has been also reported [4–5].

t-MN are characterized by high incidence of complex karyotypes, frequent abnormalities of chromosome 7 and/or 5 (monosomies and/or deletions) and *TP53* mutations in 10-30% of patients [1, 6–8]. On the other hand, somatic mutations recently identified in patients with *de novo* AML and MDS, such as those of epigenetic regulators, spliceosome machinery and *SETBP1*, are rare, with the exception of *SRSF2* [9–11]. t-ALL are frequently associated with exposure to alkylating agents and/or topoisomerase II inhibitors [5–12]. Balanced translocations involving the gene *KMT2A* at 11q23, with a high prevalence of the t(4;11)(q21;q23) translocation have been commonly reported [5–12].

Cytotoxic therapy may induce chromosomal alterations and genetic mutations in hematopoietic progenitors, leading to a high incidence of complex karyotypes. *TP53* mutations in the founding clone are major contributors to genetic instability and have also been associated to the occurrence of cytogenetic abnormalities and poor response to chemotherapy that are typical of t-MN. A different scenario has been recently depicted by Wong *et al*, who showed that the number of somatic single nucleotide variants (SNV) in t-MN was similar to that of *de novo* AML and MDS, suggesting that cytotoxic therapy does not induce genome-wide DNA damage [13]. On the other hand, using a modified next generation sequencing (NGS) protocol that allows the detection of very low *TP53* variant allele frequencies, the authors of this study found the same *TP53* mutation identified at the time of t-MN diagnosis in bone marrow (BM) samples collected prior to any chemotherapy. In this line, somatic mutations in genes associated to hematological malignancies have been found in the peripheral blood of elderly otherwise healthy individuals, suggesting that mutations can naturally occur in hematopoietic cells [14–15].

The incidence of t-MN has been increasing in the past years [16]. Although this increase can be attributed to the numerical expansion and the prolonged survival of treated patients, the identification of mechanisms explaining the underlying individual susceptibility to t-MN development could be useful to limit the risk of this complication.

The aim of our study was to investigate the role of common somatic mutations and other gene alterations during clonal evolution of 14 therapy-related leukemia patients (13 t-MN and 1 t- ALL) for whom we had collected BM samples at the time of primary diagnosis of a hematological neoplasm and/or during its follow-up. At the time of t-MN diagnosis, we studied mutations in genes belonging to epigenetic regulators (*DNMT3A*, *IDH1*, *IDH2* and *ASXL1*), spliceosome enzymes (*U2AF1*, *SF3B1* and *SRSF2*), *TP53, SETBP1, NRAS* and *KRAS*. Mutations identified in the t-MN were then tracked backwards in previous samples using high sensitivity techniques, as NGS or pyrosequencing.

## RESULTS

### Screening of genetic changes in therapy-related neoplasms

Our first aim was the genetic characterization of 13 t-MN and one t-ALL patient to identify molecular markers of the disease. Mutational screening of spliceosome machinery (*U2AF1* S34 and R35, *SF3B1* exons 13–16 and *SRSF2* exon 1), epigenetic enzyme (*IDH1* R132, *IDH2* R140 and R172, *DNMT3A* R882 and *ASXL1* exon 12), *SETBP1* (SKI homologous domain), *N-RAS* (exons 2-3) and *K-RAS* (exons 2-3) was performed using Sanger sequencing, whereas *TP53* (exons 4-9) mutations were studied by standard NGS technology. We identified 8 mutations (*IDH1* R132H, *ASXL1* Y591*, *ASXL1* S689*, *ASXL1* R693*, *SRSF2* P95H, *SF3B1* K700E, *SETBP1* G870R and *TP53* Y220C) in seven of thirteen t-MN patients (54%), whereas the t-ALL patient was *KMT2A-AFF1*-positive. No other recurrent somatic mutations in these genes were detected in the remaining 6 patients (Table 1).

**Table 1:** Patient characteristics

UPN	Age at t-MN diagnosis (yrs)	Gender	Primary Malignancy	Treatment of primary malignancy	BM-blasts (%)	Diagnosis	Latency (months)	Karyotype	Molecular marker
**1**	73	F	NHL	ProMACE-Cytabom + RT	12	t-MDS	100	46,XX [25]	*ASXL1* Y591*
**2**	62	F	APL	AIDA 2000 [17]	14	t-MDS	30	46,XX [9]/42-45, ring [13]	*TP53* Y220C
**3**	63	M	NHL	R-CHOP, R-MICMA, autoSCT; bortezomib/lenalidomide	4	t-MDS	83	47,XY,+21 [12]	*ASXL1* S689*
**4**	62	F	NHL	CHOP, MICMA, autoSCT, fludarabin	8.5	t-MDS	124	46,XX [12]/45,XX,-7 [6]	*ASXL1* R693*
**5**	30	M	NHL	CODOX-M/IVAC,MICMA, autoSCT	10	t-MDS	54	46,XY,-7 [10]	absent
**6**	63	F	NK-AML and Breast Ca	AML-12 [18], Carboplatin, taxotere, herceptin + RT	10	t-MDS	83	46,XX [24]/46,XX,del(5)(q14q34) [1]	*SF3B1*K700E
**7**	74	F	NHL	CHOP, R-FC, chlorambucil	19	t-MDS	66	46,XX [25]	absent
**8**	60	F	APL	AIDA + AutoSCT	6	t-MDS	100	46,XX,-7, +21 [11]/45,XX, -7 [2]	absent
**9**	81	M	NHL	Chlorambucil + RT	13	t-MDS	100	Not available	*IDH1* R132H*SRSF2* P95H
**10**	74	M	NHL	R-FND, R-MICMA	4.5	t-MDS	74	46,XY [19]/44,X,-7, del(1)(p35), del(5)(q13), del(11)(q14) [6]	absent
**11**	37	F	HL	BEACOPP escalated	18	t-MDS	83	47-49,XX,-7, ring [10]/46,XX [1]	absent
**12**	50	F	HL	BEACOPP escalated	43	t-AML	18	46,XX [25]	absent
**13**	43	M	ALL	GMALL 05/93 [19]	7	t-MDS	32	45,XY,-7 [6]	*SETBP1* G870R
**14**	40	F	APL	AIDA 2000 [17]	80	t-ALL	18	46,XX,t(4;11)(q21;q23)[5]	*KMT2A/AFF1*

Legend: NK: normal karyotype; RT: radiotherapy; ProMACE-CytaBOM: cyclophosphamide, doxorubicin, etoposide, bleomycin, vincristine, methotrexate and prednisone; AIDA: ATRA, idarubicine, mitoxantrone; R: Rituximab; CHOP: cyclophosphamide, adriblastin, vincristine, prednisone; MICMA: mitoxantrone, carboplatin, cytarabine, methylprednisolone; CODOX-M/IVAC: cyclophosphamide, doxorubicin, vincristine, methotrexate, etoposide, ifosfamide and cytarabine; FC: fludarabin, cyclophosphamide; FND: fludarabin, mitoxantrone, desametazone; AutoSCT: autologous stem cell transplantation; BEACOPP: bleomycin, etoposide, doxorubicin, cyclophosphamide, vincristine, procarbazine, prednisone.

The apparently low frequency of TP53 mutations identified in the present patient cohort (1 in 14 patients) may not be considered representative of t-MN in general, since it is the result of the particularly restrictive patient selection, which included only patients with a previous history of hematological malignancies, and available bone marrow samples prior to t-MN development.

### Validation and quantification of variants

**Figure 1:**
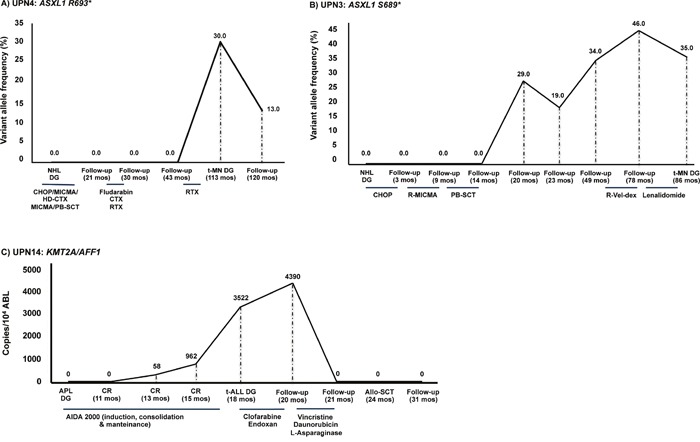
Genetic changes present in t-MN were not detectable at primary cancer diagnosis **A**. In UPN4, the *ASXL1* R693* mutation detected in the t-MN sample was undetectable at the time of NHL diagnosis (113 months prior to t-MN onset). **B**. In UPN3, the *ASXL1* S689* mutation was undetectable at the time of primary cancer diagnosis (NHL) but was detectable for the first time during NHL follow-up (at 20 months) and increased until the time of t-MN diagnosis (at 86 months). Of note, the mutation was first detected after high-dose therapy and PB-SCT. **C**. Similarly, the *KMT2A-AFF1* fusion identified in UPN 14 at t-ALL diagnosis was, as expected, undetectable in the primary APL diagnostic sample, and was detected at low levels by Q-RT-PCR thirteen months after achievement of complete molecular remission of APL, using the AIDA 2000 protocol [17]. Notably, a constant increase in the transcript copy number was evident from first identification (58 copies/10^4^ ABL) to t-ALL onset (3522 copies/10^4^ ABL). The fusion transcript became undetectable only after re-induction treatment according to the LAL0904 protocol [27] and resulted to date undetectable (54+ months) after ASCT. The variant allele frequency (VAF) is indicated. Legend. NHL DG: non-Hodgkin lymphoma diagnosis; t-MN DG: therapy-related myeloid neoplasm diagnosis; APL DG: acute promyelocitic leukemia diagnosis; CR: complete remission; t-ALL DG: therapy-related acute lymphoblastic leukemia diagnosis; Allo-SCT: allogeneic stem cell transplantation; CHOP: cyclophosphamide, adriblastin, vincristine, prednisone; MICMA: mitoxantrone, carboplatin, cytarabine, methylprednisolone; PB-SCT: peripheral blood stem cell transplantation; RTX: radiotherapy; CTX: cyclophosphamide; HD-CTX: high-dose cyclophosphamide; R-MICMA: mitoxantrone, carboplatin, cytosine arabinoside, and methylprednisolone; R-Vel-dex: lenalidomide, bortezomib, dexametasone; AIDA: ATRA, idarubicine.

**Figure 2:**
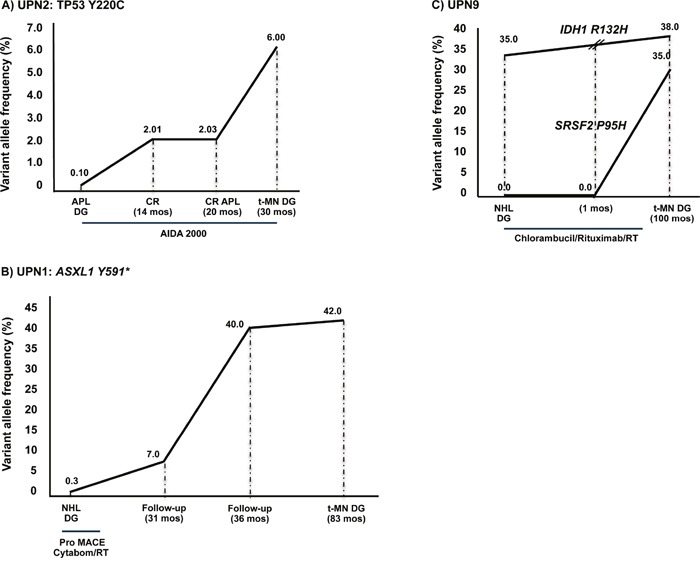
Somatic mutations were present prior to any cytotoxic treatment **A**. A *TP53* Y220C mutation was identified in UPN2 in all the available BM-MNC specimens, from APL diagnosis to complete remission, and expanded in the t-MN clone. **B**. Similarly, in UPN1 the *ASXL1* Y591* mutation was identified in all the available BM-MNC specimens, from NHL diagnosis to t-MN. Notably, this mutation was detected at very low levels in the BM NHL specimen (0.3%), significantly increased at 31 months follow-up (7%) and reached the highest VAF at t-MN diagnosis (42%). **C**. In UPN9, the *IDH1* R132H mutation was originally present at a high allele frequency at NHL diagnosis, 9 years before t-MN onset, when the patient was 72 years old, indicating that this mutation could have occurred as a pre-leukemic event. The *SRSF2* P95H mutation was acquired later, at the time of t-MN diagnosis. Legend: APL DG: acute promyelocitic leukemia diagnosis; CR: complete remission; NHL DG: non-Hodgkin lymphoma diagnosis; t-MN DG: therapy-related myeloid neoplasm diagnosis; AIDA: ATRA, idarubicine; RT: radiotherapy; ProMACE-CytaBOM: cyclophosphamide, doxorubicin, etoposide, bleomycin, vincristine, methotrexate and prednisone.

Pyrosequencing analyses, specifically designed to quantify the mutations confirmed the data obtained from Sanger sequencing in all 8 cases. Mutations identified in epigenetic regulators, spliceosome enzymes and *SETBP1* were present at a variant allele frequency (VAF) of 20 to 42% at the time of t-MN diagnosis (Figure 1), whereas the *TP53* mutation was detectable at a lower frequency (6.75%, Figure 2). Interestingly, UPN9 carried two mutations (*IDH1* R132H and *SRSF2* P95H), at a similar VAF (38% and 35%, respectively), suggesting that the two mutations were present in the same leukemic clone. The mutations were somatically acquired in the myeloid compartment, as shown by their presence also in PB-granulocytes, whereas they were not detectable in the CD3+ T-lymphocyte population.

Quantitative evaluation of the *KMT2A-AFF1* transcript in UPN 14 revealed strong positivity for the chimeric transcript at the time of t-ALL diagnosis (3500 copies/10^4^ ABL).

### Disease markers and clonal evolution

Using ultra-deep NGS, we then tracked backwards by NGS the t-MN clonal markers in BM samples collected at the time of the primary hematological malignancy, and identified at least two different patterns.

In the first scenario somatic mutations characterizing the t-MN clone were not present at primary cancer diagnosis and appeared after cytotoxic treatment. In UPN 3, UPN4, UPN6 and UPN13, the *ASXL1* S689*, *ASXL1* R693*, *SF3B1* K700E and *SETBP1* G870R mutations, studied using ultra-deep NGS, were undetectable at the time of primary cancer (NHL in UPN3 and UPN4, normal karyotype AML in UPN6 and B-ALL with a complex karyotype in UPN13) and were detected after 20, 113, 83 and 32 months from start of primary tumor treatment. In UPN4 (Figure 1A), UPN6 and UPN13 the identified mutations were detectable for the first time at t-MN diagnosis, suggesting a dominant role of these mutations in t-MN evolution. In UPN3, the *ASXL1* S689* mutation was detectable for the first time at NHL follow-up (VAF: 29%, at 20 months), and increased until the time of t-MN diagnosis (VAF: 35%, 86 months). Of note, the *ASXL1* S689* was first detected following peripheral blood stem cell transplantation (PB-SCT, Figure 1B). In UPN 6, who had two primary malignancies (AML and BC), the SF3B1 mutation may have been induced by the chemotherapy used to treat AML or by the combined radio-chemotherapy approach used to treat BC. In this case we have no data to precisely unravel the genomic effects of the different treatments.

Similarly, the *KMT2A-AFF1* fusion identified in UPN14 at t-ALL diagnosis, as expected, was undetectable in the primary APL diagnostic sample, and was detected at low levels by Q-RT-PCR 13 months after achievement of complete remission of APL, treated according to the AIDA protocol (Figure 1C, 17). Notably, a constant increase in the transcript copy number was evident from first identification (58 copies/10^4^ ABL) to t-ALL onset (3522 copies/10^4^ ABL). The patient did not achieve molecular response after induction treatment for ALL using clofarabine and cyclophosphamide (4390 copies/10^4^ ABL), whereas re-induction treatment according to the LAL0904 protocol (3 courses of Vincristine and Daunorubicin, 20) resulted in complete hematological and molecular remission. UPN14 subsequently underwent allogeneic stem cell transplantation (ASCT) from an HLA-identical related donor with T-cell depletion and is to-date in complete hematological and molecular remission 54+ months after ASCT.

A different pattern was observed in UPN1, UPN2 and UPN9, where mutations were detectable in the primary BM sample, prior to any cytotoxic treatment. In particular, the specific *TP53* Y220C mutation was identified in UPN2 in all available BM-MNC specimens, from APL diagnosis through complete remission phases, and finally in t-MN. However, the *TP53* Y220C mutation was undetectable in mesenchymal stromal cells (MSC) cultured from the BM at t-MN diagnosis, indicating that the mutation had been acquired by hematopoietic cells only. A high-throughput NGS analysis revealed a constant increase in the mutated allele frequency from APL to t-MN onset (Figure 2A).

Similarly, the *ASXL1* Y591* mutation was identified in UPN1 in all the available BM-MNC specimens, from NHL diagnosis to t-MN. Notably, *ASXL1* Y591* mutation was detected at very low level in the NHL BM-specimen (0.3%), significantly increased at 31 month follow-up (7%) and reached the highest VAF at t-MN diagnosis (42%, Figure 2B).

UPN9 was characterized by a particular pattern, with the *IDH1* R132H mutation present at NHL diagnosis, whereas the *SRSF2* P95H mutation appeared at t-MN onset (Figure 2C). In particular, the *IDH1* R132H mutation, originally present at a high allele frequency (35%) 9 years before t-MN onset, when the patient was 72 years old, indicates that this mutation could have occurred as a pre-leukemic event. The later acquisition of a *SRSF2* P95H mutation suggests its role in t-MN pathogenesis in a susceptible individual.

Altogether, these data suggest that different patterns of therapy-related leukemogenesis may occur in different patients.

## DISCUSSION

Changes in cytotoxic treatment for different malignancies have modified the incidence of t-MN [16]. Since only a small proportion of cancer patients develop a t-MN, individual susceptibility to this disease is one of the topics of major interest. In the past years, several authors have suggested that germ-line variants may play a major role in t-MN predisposition. Candidates are polymorphisms of genes implicated in detoxification, DNA repair and apoptosis pathways [21–23], or single nucleotide variants (SNV) affecting cancer predisposition genes, such as Fanconi anemia genes [7, 24]. Although next generation sequencing approaches have allowed the acquisition of detailed information, a direct and univocal mechanism to explain t-MN susceptibility has not been identified yet.

Recently, somatic mutations in critical genes, including *DNMT3A*, *ASXL1* and *TP53* have been identified in the PB of healthy elderly individuals, indicating that mutated clones may spontaneously emerge as a result of the aging process [14–15]. In this scenario, therapy-related leukemia would not be a direct consequence of cytotoxic treatment, but the result of clonal selection due to cytotoxic treatment administered to predisposed individuals.

In the present investigation, we studied the time-course of the appearance of somatic mutations in genes known to be involved in hematological malignancies during therapy-related leukemogenesis.

Our results highlight at least two different models. In a first model, mutations were acquired probably as a consequence of cytotoxic treatment, where *ASXL1* S689* (UPN3), *ASXL1* R693* (UPN4), *SF3B1* K700E (UPN6) and *SETBP1* G870R (UPN13) mutations were only detectable at the time of t-MN diagnosis, and were apparently absent in the BM-MNC harvested at time of primary tumor diagnosis and during follow-up. Although we cannot exclude the presence of minor clones (VAF <0.1%) in UPN3, UPN4, UPN6 and UPN13, our results suggest that the *ASXL1* S689*, *ASXL1* R693*, *SF3B1* K700E and *SETBP1* G870R mutations may be directly induced by chemo- and/or radiotherapy. The acquisition of these mutations at high VAF may play a pivotal role in the development of t-MN.

Similarly, in UPN14, the *KMT2A-AFF1* transcript was not amplified at APL diagnosis and was detected for the first time 14 months after APL treatment start, during maintenance treatment that was being given according to the AIDA 2000 protocol [17]. The *KMT2A/AFF1* copy number progressively increased later on, until t-ALL diagnosis. The standard AIDA protocol includes consolidation with mitoxantrone, a topoisomerase-II inhibitor, known to be involved in DNA-damage specifically targeting the *KMT2A* gene [25]. t-MN following Topo-II inhibitors are also characterized by short latency, as in this case, where the *KMT2A/AFF1*-positive clone became detectable 13 months after treatment start. The high curability of APL highlights the importance of avoiding genotoxic and mutagenic drugs in this disease, where chemotherapy-free regimens including arsenic-trioxide have shown high curative potential at least in non-high risk patients [26].

In the second scenario, somatic mutations were present in the marrow harvested at the time of primary cancer diagnosis. This is exemplified by UPN2, who carried a *TP53* Y220C mutation at a frequency of 0.1% at primary APL diagnosis, progressively increasing in serial follow-up samples, and until t-MN onset. A low, but significant number (6%), of *TP53* mutated cells may suffice to guide t-MN development. This same pattern has been previously identified by Wong et al. in four of seven t-MN patients, suggesting that cytotoxic therapy does not directly induce *TP53* mutations, but selects for hematopoietic progenitor cells carrying spontaneous *TP53* mutations, which are more resistant to chemotherapy than *TP53* wild-type cells [13]. The presence of *TP53* mutations could contribute to the typical genetic instability of t-MN, associated with complex karyotypes and poor response to chemotherapy.

The same scenario identified in UPN2 could be hypothesized in UPN1, where the *ASXL1* Y591* mutation was detected at low frequency (0.3%) at primary NHL diagnosis and progressively increased in serial follow-up samples, reaching a very high VAF at the time of t-MN diagnosis (42%).

In UPN9, we found two mutations in the t-MN sample (*IDH1* R132H and *SRSF2* P95H), but only the *IDH1* R132H was present at the time of NHL diagnosis, probably acting as predisposing factor for the acquisition of the second mutation. The similar VAF (35-38%) of the two mutations suggests their presence in the same cell clone, although we cannot exclude that two independent clones were simultaneously present. According to the literature, *IDH1* mutations are rare or absent in NHL, therefore it seems unlikely that these were present in the NHL clone in our case [27]. The high VAF of the *IDH1* mutation in the initial BM sample (35%) which remained virtually unchanged at t-MN diagnosis (38%) may indicate independence of this clone from the pressure of cytotoxic therapy with chlorambucil. Since this drug has only rarely been associated with t-MN, it remains unclear whether this was a true therapy-related MDS or a second neoplasm occurring in a susceptible patient [28]. In this line, Lindsley et al, have recently shown that mutations of splicing and epigenetic enzymes may distinguish AML subtypes which are “secondary” to MDS, from truly therapy-related forms [9].

In conclusion, our data show that secondary leukemogenesis is a heterogeneous process at least in cases with a previous hematological malignancy. Somatic mutations in critical genes may precede and favor leukemic development or may be induced by the cytotoxic treatment. Additional studies in extended patient cohorts with a longitudinal follow-up, together with further advances in NGS technology and digital PCR, are needed to define the contribution of minor clones to therapy-related leukemogenesis. This will also help to identify patients at major risk at the time of primary cancer diagnosis, where more leukemogenic treatments could be avoided and a more stringent follow-up monitoring should be recommended.

## MATERIALS AND METHODS

### Study population

Our study population included 14 patients (13 t-MN and one t-ALL), diagnosed at the Department of Hematology of the Università Cattolica del Sacro Cuore and at Tor Vergata University between November 2003 and March 2014. Cases were selected based on availability of DNA from secondary leukemia diagnosis and at least one preceding phase (e.g. marrows collected during follow-up of the primary tumor). There were 5 males and 9 females, with a median age at t-MN diagnosis of 58 years (range 30–81). According to the proportion of blasts, there were 12 MDS, 1 AML and 1 B-lineage ALL. The primary malignancies were all hematological and included non-Hodgkin lymphoma (NHL) in 7 patients, Hodgkin lymphoma (HL) in 2 patients, acute promyelocytic leukemia (APL) in 3 patients, normal-karyotype AML followed by breast cancer (BC) in 1 patient and B-ALL in 1 patient. Detailed patient characteristics are reported in Table 1. All peripheral blood (PB) and BM samples were obtained after informed consent. The study has been approved by the Institutional Ethical Committees of the Università Cattolica del Sacro Cuore and Tor Vergata University.

### Mutational analysis

Mononuclear cells (MNCs) were separated from patients’ BM at the time of the primary cancer diagnosis, during its follow-up and at t-MN diagnosis by Ficoll gradient centrifugation using Lympholyte-H (Cedarlane, Ontario, Canada). As previously reported [29], BM-mesenchymal stem cells (MSC) were expanded in UPN2 using Mesencult medium (Stem Cell Technologies, Voden Medical Instruments, Milan, Italy) in plastic-adherent cultures up to the second passage. Granulocyte, CD3+ and CD3- cells were isolated at t-MN diagnosis from the peripheral blood of UPN9 by Ficoll gradient centrifugation followed by MACS cell separation (Miltenyi biotec, Italy).

Genomic DNA was extracted using the QIAamp DNA Mini Kit (Qiagen Srl., Milan, Italy), following the manufacturer's instructions, whereas total RNA was extracted using standard procedures and reverse-transcribed using random hexamers as primers [30]. The following mutations were studied at the time of t-MN diagnosis by Sanger sequencing of genomic DNA: *IDH1* R132, *IDH2* R140 and R172, *DNMT3A* R882, *ASXL1* exon 12, *U2AF1* S34 and R35, *SF3B1* exons 13–16, *SRSF2* exon 1, *SETBP1* SKI homologous domain, *NRAS* exons 2-3 and *KRAS* exons 2-3 as previously described (ABI PRISM 3100; Applied Biosystems/Life Technologies, Milan, Italy, 10-11, 31). All mutations were confirmed in independent experiments and quantified by pyrosequencing using specifically designed oligonucleotides targeting the mutated region (shown in Supplementary Table 1). Reagents (PyroMark Gold Q96, QiagenSrl, Milan, Italy), instrumentation and analysis software used for pyrosequencing analysis were as recommended by the manufacturers (PyroMark Q96 ID, DiatechPharmacogenetics, Jesi, Italy, PyroMark Assay Design and PyroMark Q24 version 2.0.6).

*TP53* mutations were studied at the time of t-MN diagnosis by standard NGS, since mutations of this gene have been detected at low VAF by several authors at t-MN diagnosis. Briefly, genomic DNA was amplified using Fast Start High Fidelity Polymerase (Roche, Monza, Italy) and fusion primers (Roche, Monza, Italy) containing M13 adapter sequences and the sequence-specific primers (Supplementary Table 1) spanning *TP53* exons 4-9 (RefSeq NM_000546.5, representing the longer transcript encoding the longest protein isoform). Amplicon library A and B sequencing adapters and multiplex identifier (MID) tags were then added to both tails of amplicons by a second amplification step. PCR products were visualized on agarose gel, purified using AMPure XP DNA-binding paramagnetic beads (Agencourt Bioscience Corp., Beckman Coulter S.p.A, Milan, Italy), and quantified using the picogreen dye (Life Technologies, Carlsbad, California) and the Victor X2 fluorometer (Perkin Elmer, Waltham, Massachusetts). Samples were then pooled at equimolar ratios to prepare for Roche/454 pyrosequencing. The obtained amplicon library was added to the emulsion PCR at a ratio of 0.8 molecules per bead and subjected to deep sequencing on the Genome Sequencer Junior instrument (Roche-454 Life Sciences, Branford, Connecticut). The obtained sequencing reads were mapped to the *TP53* human reference sequence (RefSeq NC_000017.11) and analyzed by the Amplicon Variant Analyzer (AVA) software version 3.0 (Roche-454 Life Sciences) to establish the mutant allele frequency.

The mutations identified in the t-MN sample were then studied in marrow samples collected at the time of primary diagnosis and/or during follow-up using high-throughput NGS (Sistemas Genòmicos S.L., Valencia, Spain). Briefly, genomic DNA was amplified by PCR using specific homemade designed primers linked to specific flags (Supplementary Table 1). Amplicons resequencing was performed using the Illumina MiSeq sequencing platform and NexteraXT PCR enrichment (Illumina, San Diego, CA, USA). Paired-end’ reads of 250 nt were generated. Then, targeted regions were enriched using the Nextera XT DNA kit (Illumina, San Diego, CA, USA). For the amplicon data analysis, three different plots were generated for each sample's read group: per base sequence quality, per base sequence content and per sequence quality scores. To remove the background noise and to assure that only very confident high quality reads were used to perform read alignment, Nextera adapters were removed using FASTQ-MCF software. Reads were then collapsed using UPARSE [32] to merge the paired-end reads into a single better quality read (no mismatches allowed). Then PRINSEQ [33] was used to remove low quality reads in both 5’ and 3’ ends. Collapsed reads were aligned separately against the human reference genome version GRCh38. Read alignment was performed using BWA and ‘in-house’ scripts (Sistemas Genòmicos S.L., Valencia, Spain). From the BAM formatted file obtained after reads mapping, low quality reads were removed. In addition, the overall sample coverage and the efficiency of the combination of the selected strategy (PCR enrichment system plus NGS platform) were evaluated. Filtering processes were performed using Picard-tools (http://picard.sourceforge.net/) and SAM tools [34]. Coverage metrics and the evaluation of the target enrichment system were performed using custom scripts (Sistemas Genómicos S.L., Valencia, Spain). For each of the corresponding specific amplicon regions average coverage was above 120000x for all the samples in the experiment (detailed read depth in Supplementary Table 2). To avoid bias, for the quantification of studied mutations all collapsed mapped reads were used to establish the count and frequencies in % (alternative allele count/total depth x100).

### Detection and quantification of the KMT2A-AFF1, transcript

Qualitative screening of the t(4;11)(q21;q23) translocation resulting into the *KMT2A-AFF1* fusion transcript was performed in UPN14 at the time of diagnosis of a therapy-related B-ALL, according to van Dongen, et al. [35]. The quantitative analysis for *KMT2A-AFF1* was performed according to Gabert J, et al. [36] at the time of the primary acute promyelocytic leukemia and at various time points during its follow-up.

## SUPPLEMENTARY MATERIALS AND METHODS TABLES




