# Supplementary material for: Clonal evolution in therapy-related neoplasms
Source: Oncotarget. 2017 Jan 5;8(7):12031–40. doi: 10.18632/oncotarget.14509 (PMC5355323; doi:10.18632/oncotarget.14509)
Supplement: Supplementary file 1 [file oncotarget-08-12031-s001.pdf]

Clonal evolution in therapy-related neoplasms

SUPPLEMENTARY MATERIALS AND METHODS

For the ASXL1 NGS analysis, a mean coverage of 300.000x was used (UPN1, UPN3 and UPN4). The NGS library was paired-end (two reads R1 and R2). In this case, both reads were collapsed, and we could analyze each position twice. In the variant calling process, we only selected the reads with a global mean and local (nucleotide position) of 30 in the Phred Scale [1] [2]. This resulted in a minimum threshold of 0.001% in SNVs identification for each reads, and with two reads for each position, in a theoretical error 0,00001. In this situation, a position with 300.000 colapsed reads would give only 3 reads for random (300.000 * 0,00001). To avoid any chance of bias, our 0,1% threshold (0,05% in heterzygous variants) was much lower than our theoretical threshold 0,00001 ($p=2,10^{-16}$).

REFERENCES

1. Ewing, B. & Green, P. Base-calling of automated sequencer traces using phred. II. error probabilities. Genome research 8, 186-194 (1998).
2. Ewing, B., Hillier, L., Wendl, M. C. & Green, P. Base-Calling of automated sequencer traces using phred. I. Accuracy assessment. Genome Research 8, 175-185 (1998).

Supplementary Table 1: Primer sequences used for pyrosequencing analysis, NGS and high-throughput NGS amplicon library preparation. Specific flags linked to homemade designed primers used for High-throughput NGS are in Italic. Primers used for Sanger sequencing have been previously reported [10-11, 31].

See Supplementary File 1

Supplementary Table 2: Total sequenced reads per sample

UPN	Time points	Total reads
1	Diagnosis of primary disease (NHL)	666230
2	Diagnosis of primary disease (APL)	266930
3	Diagnosis of primary disease (NHL)	627926
4	Diagnosis of primary disease (NHL)	673560
6	Diagnosis of primary disease (NK-AML)	368574
9	Diagnosis of primary disease (NHL)	448384
13	Diagnosis of primary disease ALL)	375724
2	Follow-up (14 mos)	229130
2	Follow-up (20 mos)	227204
13	Follow-up (23 mos)	314328
