# Supplementary material for: Clonal evolution in therapy-related neoplasms
Source: Oncotarget. 2017 Jan 5;8(7):12031–40. doi: 10.18632/oncotarget.14509 (PMC5355323; doi:10.18632/oncotarget.14509)
Supplement: Supplementary file 2 [file oncotarget-08-12031-s002.docx]

**Supplementary table 1**

Primer sequences used for pyrosequencing analysis, NGS and high-throughput NGS amplicon library preparation. Specific flags linked to homemade designed primers used for High-throughput NGS are in Italic. Primers used for Sanger sequencing have been previously reported [10-11, 31].

| **Gene** | **Sequence** | | **Method** |
| --- | --- | --- | --- |
| TP53 exon 4 | Fw | 5’-CCTGGTCCTCTGACTGCTCT-3’ | NGS |
|  | Rv | 5’-TTCTGGGAAGGGACAGAAGA-3’ |  |
| TP53 exon 4 | Fw | 5’-GTCCAGATGAAGCTCCCAGA-3’ |  |
|  | Rv | 5’-GCCAGGCATTGAAGTCTCAT-3’ |  |
| TP53 exon 5 | Fw | 5’-TCTGTCTCCTTCCTCTTCCTACA-3’ |  |
|  | Rv | 5’-AACCAGCCCTGTCGTCTCT-3’ |  |
| TP53 exon 6 | Fw | 5’-CAGGCCTCTGATTCCTCACT-3’ |  |
|  | Rv | 5’-GCCACTGACAACCACCCTTA-3’ |  |
| TP53 exon 7 | Fw | 5’-CCTGCTTGCCACAGGTCT-3’ |  |
|  | Rv | 5’-GTGATGAGAGGTGGATGGGT-3’ |  |
| TP53 exon 8 | Fw | 5’-GGGAGTAGATGGAGCCTGGT-3’ |  |
|  | Rv | 5’-GCTTCTTGTCCTGCTTGCTT-3’ |  |
| TP53 exon 9 | Fw | 5’-AAAGGGGAGCCTCACCAC-3’ |  |
|  | Rv | 5’-TGTCTTTGAGGCATCACTGC-3’ |  |
| IDH1 R132H | Fw | 5’-CAAAAATATCCCCCGGCTTG-3’ | Pyrosequencing |
|  | Rv | 5’-CAACATGACTTACTTGATCCCC-3’ |  |
|  | Seq | 5’-ACCTATCATCATAGGT-3’ |  |
| SF3B1 K700E | Fw | 5’-GTGTTTGGTTTTGTAGGTCTTGTG-3’ |  |
|  | Rv | 5’-CAATGGCCAAAGCACTGA-3’ |  |
|  | Seq | 5’-TTGTGGATGAGCAGC-3’ |  |
| SETBP1 G870R | Fw | 5’-AGTCCCACAGTGAGGAGACG-3’ |  |
|  | Rv | 5’-CGCTTGGTCAGAAGTGCTGT-3’ |  |
|  | Seq | 5’-GCTGTTGTTGTCTGTCC-3’ |  |
| SRSF2 P95H | Fw | 5’-AAGCGCGACGCTGAGGAC-3’ |  |
|  | Rv | 5’-TCCCCTCAGCCCCGTTTAC-3’ |  |
|  | Seq | 5’-CGGCTGTGGTGTGAG-3’ |  |
| ASXL1 Y591* | Fw | 5’-CCTGGGTGGTTAAAGGTCAGC-3’ |  |
|  | Rv | 5’-CTTTAATGTCTGCGAGGGTCCTG-3’ |  |
|  | Seq | 5’TCCGGGGGCATATCT-3’ |  |
| ASXL! S689*-R693* | Fw | 5-GAGCACCCCTGGAAAGTGTA-3’ |  |
|  | Rv | 5’-CTGGGTATGCTCCCCATTTA-3’ |  |
|  | Seq | 5’-CCCTGGAAAGTGTACG-3’ |  |
| TP53 Y220C | Fw | 5’-GCGTGTGGAGTATTTGGATGAC-3’ |  |
|  | Rv | 5’-CTTAACCCCTCCTCCCAGAGA-3’ |  |
|  | Seq | 5’-AGACCTCAGGCGGCT-3’ |  |
| SETBP1 G870R | Fw | 5‘-*TCGTCGGCAGCGTCAGATGTGTATAAGAGACAG*ATTG  GCTCCCTAAAGGAAATC-3' | High-throughput NGS |
|  | Rv | 5’-*GTCTCGTGGGCTCGGAGATGTGTATAAGAGACAG*TCA  GAAGTGCTGTTGTTGTC-3’ |  |
| SRSF2 P95H | Fw | 5’-*TCGTCGGCAGCGTCAGATGTGTATAAGAGACAG*AAGC  GCGACGCTGAGGAC-3’ |  |
|  | Rv | 5’-*GTCTCGTGGGCTCGGAGATGTGTATAAGAGACAG*TCC  CCTCAGCCCCGTTTAC-3’ |  |
| ASXL1 Y591* | Fw | 5’T*CGTCGGCAGCGTCAGATGTGTATAAGAGACAG*CCTGGGTGGTTAAAGGTCAGC-3’ |  |
|  | Rv | 5’*GTCTCGTGGGCTCGGAGATGTGTATAAGAGACAG*CTTTAATGTCTGCGAGGGTCCTG-3’ |  |
| ASXL1 S689*-R693* | Fw | 5’*TCGTCGGCAGCGTCAGATGTGTATAAGAGACAG*GAGCACCCCTGGAAAGTGTA-3’ |  |
|  | Rv | 5’*GTCTCGTGGGCTCGGAGATGTGTATAAGAGACAG*CTGGGTATGCTCCCCATTTA-3’ |  |
| TP53 Y220C | Fw | 5’-*TCGTCGGCAGCGTCAGATGTGTATAAGAGACAG*GCGT  GTGGAGTATTTGGATGAC-3’ |  |
|  | Rv | 5’-*GTCTCGTGGGCTCGGAGATGTGTATAAGAGACAG*CTT  AACCCCTCCTCCCAGAGA-3’ |  |
| SF3B1 K700E | Fw | 5’-*TCGTCGGCAGCGTCAGATGTGTATAAGAGACAG*GTGT  TTGGTTTTGTAGGTCTTGTG-3’ |  |
|  | Rv | 5’-*GTCTCGTGGGCTCGGAGATGTGTATAAGAGACAG*CAA  TGGCCAAAGCACTGA-3’ |  |
